# Assessment of Chitosan Coating Enriched with Free and Nanoencapsulated *Satureja montana* L. Essential Oil as a Novel Tool for Beef Preservation

**DOI:** 10.3390/foods11182733

**Published:** 2022-09-06

**Authors:** Natalija Đorđević, Ivana Karabegović, Dragoljub Cvetković, Branislav Šojić, Dragiša Savić, Bojana Danilović

**Affiliations:** 1 Faculty of Technology, University of Niš, Bulevar Oslobođenja 124, 16000 Leskovac, Serbia; 2 Faculty of Technology, University of Novi Sad, Bulevar cara Lazara 1, 21000 Novi Sad, Serbia

**Keywords:** nanoparticles, meat storage, meat safety, lipid oxidation, microbial stability

## Abstract

The effect of chitosan coating enriched with free and nanoencapsulated *Satureja montana* L. essential oil (EO) on microbial, antioxidant and sensory characteristics of beef was analyzed. Different concentrations of free *Satureja montana* L. EO (SMEO) and nanoparticles (CNPs) were added to chitosan coatings, namely 0.25%, 0.5% and 1%. The beef samples were immersed in the chitosan coatings and stored at +4 °C for 20 days. In this period, the changes in pH value, total viable count (TVC), lactic acid bacteria, psychrophilic bacteria and *Pseudomonas* spp. were analyzed. The lipid oxidation of beef was determined by the TBAR assay, while sensory analysis was performed by means of the descriptive evaluation method. Generally, the influence of chitosan coating with CNPs on the growth of the tested microorganisms was more pronounced compared to SMEO. Treatment with coating enriched with 1% CNPs resulted in the reduction in TVC and *Pseudomonas* spp. by 2.4 and 3 log CFU/g, compared to the control, respectively. Additionally, all applied coatings with SMEO and CNPs resulted in the prolonged oxidative stability of the meat The addition of free SMEO created an unnatural aroma for the evaluators, while this odor was neutralized by nanoencapsulation. The durability of color, smell and general acceptability of beef was significantly increased by application of chitosane coatings with the addition of SMEO or SMEO-CNPs, compared to the control. This research indicates the potential application of enriched chitosan coatings in beef preservation in order to improve meat safety and prolong shelf-life.

## 1. Introduction

The preservation of highly perishable foods, including meat and meat products, is a persistent challenge for the industry [1]. In addition to the application of low temperatures, the most common preservation method in the meat industry is the use of synthetic preservatives, such as nitrite, benzoate, sulfite, sorbate, as well as synthetic phenolic compounds, which are known for their long-term and dangerous effects on consumers’ health [2]. On the other hand, many microorganisms have developed resistance to the action of the permissible concentrations of synthetic preservatives [3]. For these reasons, there is a justified demand from consumers, as well as science and the meat industry, for screening and application of natural, safe and effective preservatives [4].

Chitosan has attracted special attention from researchers as a natural preservative due to its great potential for the maintenance of meat quality and safety [5,6,7]. Due to its natural origin and good antimicrobial and antioxidant potential, chitosan can be used in food biopreservation in the form of films and coatings [8]. Chitosan films and coatings can incorporate active components, such as plant essential oils (EOs), which can improve their biological activity. The EOs of most plants (oregano, cinnamon, clove, thyme) are categorized as Generally Recognized as Safe (GRAS) and effective in low concentrations [9].

Chitosan coatings enriched with EOs can provide good antimicrobial and antioxidant protection and prolong the shelf life of meat [10]. However, the use of EOs in the free form is limited due their dissolution in the aqueous phase, causing their loss during storage. The possible interaction of EOs’ active compounds with meat components can reduce or completely inhibit their antimicrobial potential. In addition, due to the presence of some aromatic compounds, some EOs can have a negative impact on the sensory characteristics of the meat and meat product [11].

The nanoencapsulation of EOs aims to overcome such problems, i.e., masking unwanted odors or tastes of EOs by coating the EO inside the inert material, isolating it and preventing direct contact with food ingredients [12]. Nanoencapsulation can increase EOs’ oxidative stability, reducing the negative impact of light, moisture and high temperatures, and thus keeping their biological potential constant [13]. On the other hand, the nanoencapsulation of EOs favorably affects the physical, chemical and mechanical properties of chitosan films and coatings [14].

*Satureja montana* L. is an aromatic plant, 20–30 cm high, which grows in dry, warm and rocky areas of the Mediterranean [15] and the Balkans [16]. The genus *Satureja* includes more than 200 species of annual or perennial aromatic plants belonging to the family *Lamiaceae* [17]. The seeds, leaves, flowers and stems of this genus’ members are used in alternative medicine as an analgesic, anti-inflammatory and therapeutic agent [18]. Aromatic *Satureja montana* L. EO has proven antimicrobial [19] and antioxidant [20] activity, with great potential for application in meat preservation [21].

Maintaining the microbiological and oxidative stability of meat and meat products primarily depends on the preservation method. The application of traditional methods usually implies the use of synthetic preservatives, regardless of their possible harmful effects. Therefore, research is increasingly directed towards the application of preservatives from natural sources and their impact on the quality and sustainability of meat. So, this research aimed to evaluate the biological activity of chitosan coating enriched with free and nanoencapsulated *Satureja montana* L. EO in raw beef during storage. For this purpose, the microbiological and oxidative stability of meat was monitored, together with sensory attributes of meat during storage, as important parameters for the potential application of new method for raw beef preservation.

## 2. Materials and Methods

### 2.1. Chitosan Coating Preparation

Chitosan coatings were prepared according to the method of Pabast et al. [22], with some modifications. The chitosan solution (2%) was made by dissolving medium molecular weight chitosan powder (Sigma-Aldrich, St. Louis, MO, USA) in 1% acetic acid (>99.8%, Centrohem, Stara Pazova, Serbia) by stirring with a magnetic stirrer (SCILOGEX SCI280-Pro, Rocky Hill, CT, USA) for 2 h at 95 °C. After filtering the resulting solutions, 0.25 mL/g of glycerol (98+%, Fisher Scientific, Loughborough, UK) was added to the chitosan solution. Stirring was continued for 30 min at room temperature. Thereafter, a surfactant, Tween 60 (Fisher Scientific, Loughborough, UK), at a concentration of 0.1% was added and stirring was continued at 60 °C for 30 min. After cooling to room temperature, *Satureja montana* L. essential oil (SMEO) or nanoparticles in concentrations of 0.25%, 0.5% and 1% was added to the chitosan coating.

### 2.2. Preparation of Chitosan Nanoparticles Loaded with SMEO

Raw plant material (*Satureja montana* L.) was collected from Rtanj mountain (1565 m a.s.l, 43°46′34″ N 21°53′36″ E), near Sokobanja, Serbia, and identified by the authors [23]. Harvesting was performed in the flowering period during August and September. The SMEO was obtained by means of microwave-assisted extraction from air-dried plants. The chemical composition of SMEO determined by gas chromatography/mass spectroscopy (GC/MS) and gas chromatography/flame ionization detection (GC/FID) analysis indicated the presence of 68 compounds, representing 97.8% of the total EO composition. The main components of SMEO were aromatic compounds (46.6%), oxygen containing monoterpenes (24.0%), sesquiterpene hydrocarbons (10.7%) and monoterpene hydrocarbons (8.2%) [23]. After extraction, SMEO was stored at 4 °C until use. Chitosan nanoparticles (CNPs) with the addition of SMEO were prepared by the ionic gelling method according to the study of Zhang et al. [24]. The chitosan solution (0.4%) was created by adding chitosan powder into 50 mL of 1% acetic acid, with constant mixing by a magnetic stirrer. Then, the solution was filtered to separate insoluble parts, after which the filtrate pH value was set to 4.7–4.8. Then, Tween 60 was added at a concentration of 0.1% and stirred at a temperature of 60 °C until a homogeneous solution was obtained. SMEO (200 μg) dissolved in 4 mL of ethyl alcohol (96%) was added dropwise to the chilled chitosan solution with stirring at 1200 rpm for 30 min. After homogenization at room temperature, 30 mL of the sodium-tripolyphosphate solution (>96%, Centrohem, Stara Pazova, Serbia) (1.87 mg/mL, pH = 4) was added in drops and stirred for 60 min. The nanoparticles loaded with SMEO (SMEO-CNPs) were obtained by centrifugation of the resulting solution at 14,000 rpm for 20 min (Eppendorf 5418 Centrifuges, Eppendorf, Hamburg, Germany) and stored at +4 °C until use.

### 2.3. Determination of Encapsulation Efficiency

To determine the efficiency of encapsulation, 200 μL of CNPs with incorporated SMEO was added to 5 mL of a 2 mol/L solution of HCl, followed by incubation in a boiling water bath for 30 min. After cooling, 2 mL of ethanol (96%) was added, and the mixture was centrifuged at 9000 rpm for 5 min. Then, the absorption spectrum of the supernatant was recorded on spectrophotometer (2100 UV spectrophotometer, Cole-Parmer, IL, USA) in the wavelengths range from 200 nm to 400 nm, with the maximum absorption wavelength of 275 nm. The encapsulation efficiency was calculated according to the following equation [24]:

$$Encapsulation\;efficiency\left(EE,\%\right)=\frac{weight\;of\;loaded\;SMEO}{weight\;of\;initial\;SMEO}\cdot 100$$

### 2.4. Nanoparticles Characterization

The determination of the characterization parameters of chitosan CNPs and SMEO-CNPs, i.e., Z-average and polydispersity index (PDI), was performed by means of the dynamic light scattering (DLS) method on the Zeta sizer 7.11 (MAL1031041, Malvern Instruments Ltd., Malvern, UK).

### 2.5. Preparation of Meat Samples

Raw beef tenderloin (48 h post-mortem) was purchased from a local butcher, and its chemical composition was determined by means of ISO standard methods: 63.0% moisture [25], 19.73% proteins [26], 5.35% total fat [27], and 1.82% ash [28]. Sample preparation was performed according to Lekjing et al. [29], with some modifications. The surface of raw beef tenderloin, cut into pieces (50 ± 1 g), was first sterilized with a UV lamp (UVSL 2055A, ENC, Belgrade, Serbia) on both sides for 10 min. Samples treated this way were immersed in a 2% chitosan coating with the addition of SMEO and SMEO-CNPs for 3 min. All treatments were performed in duplicate, and so a total of 14 samples were prepared for each treatment. Thereafter, the samples were dried under aseptic conditions for 15 min at room temperature. After drying, the meat samples were packed in sterile bags (Whirl-Pak^®^ Sample Bag, 384 mL, Madison, Wisconsin) and stored at +4 ± 1 °C in an incubator (FTC 90I, VELP Scientifica SRl, Usmate, Italy) for 20 days. Microbiological and other analyses were performed in triplicate at time intervals of 0, 2, 4, 8, 12, 16 and 20 days. Table 1 summarize the description of meat samples treatments with different chitosan coatings.

### 2.6. Microbiological Analysis of the Meat Samples

For microbiological analysis, 25 g of the meat sample was added to 225 mL of sterile saline peptone water (NaCl 0.8 g/L and peptone 1 g/L) and mixed thoroughly. An aliquot of 1 mL of appropriate dilution was transferred to a sterile Petri plate, and overlaid with an appropriate selective nutrient medium. The total viable count (TVC) and psychrophilic bacteria were determined on nutrient agar (“Torlak”, Belgrade, Serbia), with incubation at 30 °C and 7 °C, respectively. The number of lactic acid bacteria (LAB) was determined on a selective Lactobacillus MRS agar (HiMedia Laboratories, Kampenhout, Belgium) at a temperature of 30 °C. Members of the genus *Pseudomonas* spp. were determined on Pseudomonas CFC/CN agar (Merck, New Jersey, NJ, USA). After incubation, the grown colonies were counted, and the decimal logarithm was calculated from the obtained results, so they are presented as log CFU/g. All experiments were performed in triplicate.

### 2.7. Lipid Oxidation

Determination of lipid oxidation in meat was performed using the thiobarbituric acid reactive substance (TBAR) assay, as described by Zhang et al. [24]. Ten grams of a finely chopped meat sample was homogenized with 30 mL of trichloroacetic acid (Fisher Scientific, Loughborough, UK) (7,5%) for 2 min on 6000 rpm. Then, the suspension was filtered, and 5 mL of 20 mmol/L thiobarbituric acid (ISOLAB Laborgeraete GmbH, Wertheim, Germany) was added to the filtrate. The mixture was incubated at 95 °C during 30 min, cooled to room temperature, and absorbance was measured at 532 nm. The standard curve of 1,1,3,3-tetramethoxypropane, i.e., malonaldehyde (MDA) (Sigma-Aldrich, St. Louis, MO, USA), was obtained by dissolving a 10 mM solution in a ratio of 1 to 500 in distilled water. All analyses were performed in triplicate and the results of the TBAR test are shown as mg MDA/kg of meat.

### 2.8. Determination of pH Value

For pH value determination, 10 g beef samples were added into 100 mL of distilled water and stirred for 15 min. After homogenization, the pH value was measured using a pH-meter (HANNA HI 9318, Leighton Buzzard, UK).

### 2.9. Sensory Analysis

The descriptive evaluation of beef samples was performed by seven semi-trained evaluators from the Department of Food Science familiar with the characteristics to be analyzed. Sensory analysis of beef samples included the descriptive evaluation of sensory characteristics such as “color”, “odor” and “overall acceptability” using a 5-point descriptive scale. A score of 5 was declared as “extremely acceptable”, while a score of 1 was deemed “extremely unacceptable” regarding a property. The sensory characteristics of the samples with a score less than 3 were considered as below the acceptable level [24].

### 2.10. Statistical Analysis

The experiments were performed in triplicate and the results were presented as average value ± standard deviation. One-way ANOVA followed by Tukey’s multiple comparison test were used for the calculation of the statistical difference between the results in the software SPSS 21.0 trial version (IBM, Armonk, NY, USA). Comparison was performed considering treatments as study factors in one case, and storage days in another. Samples were considered significantly different when the *p*-value was lower than 0.05.

## 3. Results and Discussion

### 3.1. Nanoparticles Characterization

The characterization parameters of CNPs and SMEO-CNPs are shown in Table 2. The efficiency of SMEO nanoencapsulation within the chitosan matrix in this work was 63.2 ± 2.2%.

*Satureja khuzentanica* EO in the lecithin:cholesterol system had a similar nanoencapsulation effectiveness (69%) [22]. On the other hand, the encapsulation efficiency of other EOs in the chitosan system was lower. Thus, the encapsulation efficiency of Paulownia Tomentosa EO was 40.6% [24], and for tarragon EO, it was 35% [30]. In contrast, the encapsulation efficiency of *Cuminum cyminum* L. EO in lecithin:cholesterol system reached 75% [31].

The size of the particles represents one of the main parameters affecting the ability to release encapsulated agents from the particles [31]. The average size of the nanoparticles, i.e., the Z-average values for CNPs and SMEO-CNPs, was 415.4 nm and 139.9 nm, respectively. A similar value was recorded in Cuminum cyminum L. EO NPs, namely 140 nm [31]. Higher Z-average values were observed in Zataria multflora EO NPs and Benium persicum EO NPs, of 506 nm and 455 nm, respectively [32]. The Z-average value for Satureja khuzestanica NPs was lower, at about 96 nm [22]. The average particle size can be influenced by several parameters, such as pH, temperature, method of mixing, degree of deacetylation of chitosan, and ratio of chitosan and tripolyphosphate [33].

The PDI represents a measure of the homogeneity of the nanoparticle distribution, where a value closer to 0 indicates a homogeneous distribution of particles, while a value closer to 1 indicates a heterogeneous distribution [32]. In this work, the PDI indexes for CNPs and SMEO-CNPs were 0.718 and 0.241, respectively, indicating a bimodal distribution of particles. According to the results, a homogeneous distribution of particles can be observed in SMEO-CNPs, in contrast to CNPs. Other literature data showed a higher PDI index of CNPs encapsulating different essential oils [22,24,31,32].

### 3.2. Microbial Analysis

#### 3.2.1. Total Viable Count

The change in total viable count (TVC) in meat samples during the storage period is shown in Table 3. The initial number in all samples was in the range from 2.34 to 2.46 log CFU/g. As expected, from the second day of storage, the TVC in control samples was significantly higher compared to treated samples (*p* < 0.05).

The treated samples showed a significant reduction in TVC during the observed period. The use of CH reduced the final TVC by almost 0.7 log CFU/g compared to the control sample. With the use of CH-SMEO, there was a significant reduction in TVC compared to the control sample and CH. However, the most pronounced antimicrobial activity was observed in CH-SMEO 1% samples, starting from the 8th day of storage, with a significant difference compared to lower concentrations (*p* < 0.05). However, the use of CH-SMEO-CNPs in all concentrations had a stronger antimicrobial effect. At the end of the storage period, a significant trend in the reduction in TVC was observed, both among the CH-SMEO-CNP samples and compared to all other samples. The application of 1% CH-SMEO-CNPs led to a reduction in TVC by approximately 2.5 log CFU/g compared to the control sample. Since meat containing a bacterial count of less than 7 log CFU/g is considered fresh and safe for use [30], meat samples treated with CH-SMEO-CNPs may be considered acceptable, even after 20 days of storage.

Other EOs, such as *Cuminum cyminum* L. EO, included in the chitosan coating at a concentration of 1%, reduced TVC by 3 log CFU/g compared to the control sample [34]. Good TVC reduction by the application of a chitosan coating and CNPs with the addition of *Satureja khuzsestanica* EO in lamb has also been observed [22]. Namely, with the use of free oil and nanoparticles, the reduction in TVC compared to the control at the end of the 20th day of storage was 1 log CFU/g and 2 log CFU/g, respectively [22]. The addition of cinnamon EO to CNPs also had a better effect on reducing TVC (4 log CFU/g) compared to free cinnamon EO (3.5 log CFU/g) in beef after a storage period of 8 days [35].

The effectiveness of the antimicrobial action of a plant’s EO depends on its chemical composition and the ratio of active components [36]. The complex chemical composition of *Satureja montana* L., with the highest percentage of *p*-cymene (9.6%), limonene (2.4%), carvacrol (18.3%), thymol (12.5%) and germacrene D (2.5%) [23], is probably responsible for the good antimicrobial potential of SMEO and can explain the good trend in TVC reduction. Additionally, the presence of trace compounds can have a synergistic connection with the main compounds, increasing the antimicrobial potential of EOs [37]. More than 50 different compounds, most of which are aromatic, are probably responsible for the pronounced activity against TVC and other target groups of microorganisms [23].

#### 3.2.2. *Pseudomonas* spp.

The initial number of *Pseudomonas* spp. in beef samples was, on average, 2.3 log CFU/g (Table 4). Since it is known that members of the genus Pseudomonas mostly participate in the spoilage of aerobically stored meat [38], from the 4th day of storage the number increases significantly (*p* < 0.05) and reaches a value of almost 7.8 log CFU/g.

The use of a CH-SMEO with concentrations of 0.5% and 1% decreased the number of *Pseudomonas* spp. at the end of storage, but without a statistically significant difference (*p* > 0.05). Various EOs had a beneficial effect on the growth control of *Pseudomonas* spp., namely *Mentha pulegium* and *Berberis vulgaris* [39], as well as tarragon EO [30] in different types of meat. A significant difference in the change in *Pseudomonas* spp. number was observed in CH-SMEO-CNP-treated samples (*p* < 0.05) (Table 4). The reduction in bacterial number for CH-SMEO-CNP concentrations of 0.25%, 0.5% and 1% was 2.13, 2.55 and 3.02 log CFU/g at the end of observed period, respectively. Apparently, CH-SMEO-CNPs had a better antimicrobial effect compared to free EO. Similarly, antimicrobial activity was recorded in fresh turkey fillets by using CNPs incorporated with *Zataria multiflora* and *Benium persicum* EOs, reducing the *Pseudomonas* spp. number by about 2 log CFU/g after the 18th day of storage [32]. CNPs combined with *Satureja khuzestanica* EO had a pronounced effect on this bacterium, reducing the final value by 5 log units compared to the control sample [22].

Variations in the antimicrobial action of various EOs as free or incorporated into different matrices depends on the type of plant, growing conditions, extraction methods, chemical composition and method of application [40]. The use of EOs in the free state in chitosan coating causes their easy degradation by evaporation or chemical communication between meat proteins and EO components [11], thus reducing the antimicrobial potential. Nanoencapsulation preserves the stability of EOs, releasing them in a controlled manner over a longer period of storage, which explains the prolonged microbial stability of meat samples treated with CNPs compared to free EOs [14].

#### 3.2.3. Lactic Acid Bacteria

Some species of LAB are part of the natural population of raw meat. Due to the production of metabolites, such as lactic acid, meat can be protected from microbial spoilage. However, uncontrolled LAB growth leads to structure disorders, mucus and an unpleasant odor [41]. Compared to other analyzed groups of microorganisms, the initial number of LAB in meat samples was lower, approximately 1.4 log CFU/g (Table 5).

During the storage period, there was a constant increase in population in the control samples, so on the 20th day it reached a value of 6.35 log CFU/g. From the 4th day, the number of LAB in beef samples treated with CH-SMEO-CNPs was significantly reduced, compared to other samples (*p* < 0.05). At the end of storage period, the number of LAB in the samples treated with 1% CH-SMEO-CNPs was reduced by 2.54 log CFU/g. There was no significant difference in the reduction in the LAB number in CH-SMEO 0.5% and 1%, as well as 0.25% and 0.5% CH-SMEO-CNPs (*p* > 0.05).

Other studies showed a higher initial number of LAB in turkey meat (3.5 log CFU/g) samples and lower antimicrobial potential of chitosan coating with addition of *Mentha pulegium* and *Berberis vulgaris* EOs (about 2 log CFU/g) [39]. Chitosan coating with the addition of *Origanum vulgare* EO also had a low activity against LAB in turkey (0.5 log CFU/g) [42]. However, chitosan-based NPs with the addition of cinnamon EO in beef had a significant effect on reducing the LAB population (2 log CFU/g) [35]. The same level of reduction was observed with the use of CNPs with the addition of *Zataria multiflora* and *Benium persicum* EOs in turkey meat [32] and *Cuminum cyminum* L. EO in sardine fillets [31]. Other studies also showed a stronger antimicrobial potential of nanoencapsulated EO against LAB population compared to free EO [22,24].

#### 3.2.4. Psychrophilic Bacteria

Although storage at low temperatures in one of the ways to preserve meat and prevent further spoilage [43], the growth and reproduction of psychrophilic bacteria leads to violation of microbiological safety of raw meat at storage temperatures [44]. The change in the number of psychrophilic bacteria in beef samples is shown in Table 6.

In the control sample, the number of psychrophilic bacteria increased constantly during the storage period, with a significant difference (*p* < 0.05). The chitosan coating itself had good antimicrobial activity, decreasing the number of psychrophilic bacteria by almost 0.6 log CFU/g. Comparing CH-SMEO samples, a significant reduction was caused by the application of a concentration of 1%. However, the application of CH-SMEO-CNPs in all three concentrations showed the strongest antimicrobial activity with a pronounced difference compared to other samples. Thus, the reduction in the number of psychrophilic bacteria in the 1% CH-SMEO-CNP sample was approximately 2.49 log CFU/g at the end of the observed period.

A similar level of reduction in the psychrophilic bacteria number was observed with the use of chitosan solution with the addition of 1% *Cuminum cyminum* L. EO, where after 15 days of storage, the initial number was reduced by 3 log units [34]. On the other hand, there was no difference in the decrease in this number with the application of free and nanoencapsulated *Zataria multiflora* and *Benium persicum* EOs in turkey [32].

### 3.3. pH Value

During meat storage, although there is a constant increase in the number of LAB in meat samples (Table 5), the pH value increased as a consequence of the protein decomposition process, leading to the formation of sulphide compounds, amines and ammonia, [45,46]. The change in the pH value of analyzed beef samples during storage is shown in Table 7.

The initial pH value of the meat samples was 5.24 to 5.49. The recorded lower initial value of pH in the treated samples, compared to the control, is probably due to the content of 1% acetic acid in the chitosan coating. The pH value of raw beef strongly depends on the postmortem period [47]. In the first 48 h of the postmortem period, the pH value decreases to 5.4–5.7 [48], which is in agreement with the initial values recorded in this research. In the control sample, as the storage period increases, the pH value increased constantly. A significant leap in pH value was recorded after the 8th day for all samples (*p* < 0.05). The growth of pH value in CH samples did not differ significantly from the control until the 12th day of storage. Meat samples with a pH value over 5.8 are considered unsuitable for use [49]. The control exceeded the defined upper limit on the 4th day of storage, while the CH-SMEO 0.25% and 0.5% samples exceeded this limit on 8th day of storage. Treatments with CH-SMEO-CNPs in the same concentrations as CH-SMEO resulted in extending of the shelf life of beef samples by 4 days, most likely due to the longer release of the active components of SMEO, which contributes to the preservation of the meat stability [13]. Thus, at the end of the observed storage period, there was a significant difference in pH values, which were 6.71 and 6.29 for CH-SMEO 1% and 1% CH-SMEO-CNPs, respectively.

Other studies have reported a beneficial effect of EO addition on controlling the increase in pH value in meat. In turkey meat, using free *Mentha pulegum* [39] and *Cuminum cyminum* L. [34] EOs, no change in the pH value was recorded during 20 and 15 days of storage, respectively, at +4 °C. Furthermore, the difference in the effect of applied free and nanoencapsulated *Satureja khuzestanica* EO on the pH value in lamb was recorded, with final values 6.4 and 6.2, respectively [22].

Differences in the pH value variations may be explained by protein degradation [22] and the growth of the microbial population during storage (Table 3, Table 4, Table 5 and Table 6). Namely, an increase in the pH value has a favorable effect on the growth of the microbial population, while on the other hand, microbial enzymes support the decomposition of low-molecular mass compounds (such as amino acids), which further increases the pH value of meat [46]. In addition to the temperature, the pH value is an important parameter in the sustainability of the physical properties of the meat, such as softness, ability to retain water and color [50].

### 3.4. Lipid Oxidation

One of the most common causes of meat spoilage during storage, in addition to microbial factors, is the lipid oxidation of meat [51]. This can be caused by lipid hydrolysis or the oxidation of polyunsaturated fatty acids [4]. The level of lipid oxidation is expressed as the amount of MDA, the main stable oxidation product of polyunsaturated fatty acids [52]. Table 8 shows the TBARS assay data, i.e., the MDA content in meat samples during storage.

The initial content of MDA in beef samples at the beginning of storage was in the range of 0.27–0.32 mg MDA/kg of meat. From the 2nd day of storage, in the control sample, the concentration of MDA doubled and continued to continuously increase to the final value of 3.26 mg MDA/kg. There was no significant difference between the control sample and the CH sample until the 8th day, while there was a significant difference compared to other treated samples (*p* < 0.05). From the 4th day in CH-SMEO treated samples, there was no significant difference in the reduction in MDA concentration, regardless of the applied concentration. The exception was at the end of storage, where the CH-SMEO 0.25% sample did not differ from the control sample. By applying CH-SMEO 1%, the content of MDA was reduced by almost 3 times at the end of the observed period (1.10 mg/kg). Application of CH-SMEO-CNPs showed better antioxidant activity, among which the most pronounced was in the sample with the highest concentration, i.e., 1% CH-SMEO-CNPs.

The upper limiting concentration of MDA in meat is not defined according to any legislation; however, values over 2 mg/kg indicate the existence of certain oxidative changes in meat, and the appearance of rancidity can be noticed sensorily [53]. Bearing this fact in mind, it can be concluded that the treatment with 1% CH-SMEO-CNPs had the greatest contribution to maintaining the oxidative stability of beef until the end of storage. At the end of the observed storage period, the value of MDA content in the same sample was approximately 4.7 times lower compared to the control sample.

Application of other EOs added to the chitosan coating showed good antioxidant activity [39,42,54]. Other comparative studies showed a significant difference in antioxidant activity using free and nanoencapsulated EOs. Thus, the application of *Satureja khuzestanica* EO and CNPs reduced the MDA content to 3.5 mg/kg and 1.5 mg/kg, respectively, in lamb meat on the 20th day of storage [22]. Somewhat lower antioxidant activity was recorded with the application of free and nanoencapsulated *Paulownia Tementosa* EO, where there was a decrease in MDA in pork chops by 0.8 and 1 mg/kg, respectively, compared to the untreated sample [24]. Good antioxidant activity in beef was demonstrated by the use of cinnamon CNPs, where after the 8th day of storage, a very low level of MDA (0.3 mg/kg) was recorded [35]. In addition, the use of free and nanoencapsulated EOs has been shown to be successful in reducing the risk of lipid oxidation in the fresh meat of different fish species [31,55].

### 3.5. Sensory Analysis

**Figure 1:**
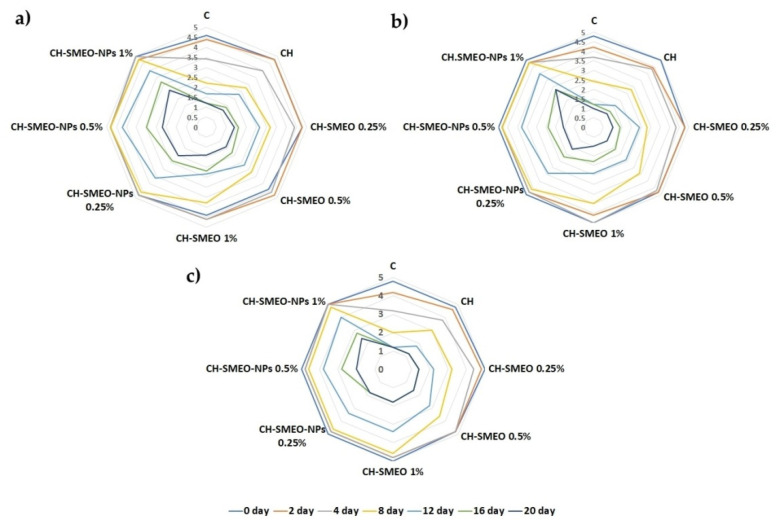
Sensory evaluation of beef samples treated with chitosan coatings and chitosan-based nanoparticles enriched with *Satureja montana* L. essential oil during storage: (**a**) odor; (**b**) color; (**c**) overall acceptability. CH—chitosan coating; CH-SMEO—chitosan coating enriched with of *Satureja montana* L. essential oil in different concentrations; CH-SMEO-CNPs—chitosan coating enriched with different concentrations of chitosan-based nanoparticles loaded with *Satureja montana* L. essential oil; C—control sample.

Sensory analysis of foods has become a crucial parameter in the validation of new products, through the evaluation of organoleptic properties using the senses (smell, color, taste, texture), on which the acceptance of the product by consumers depends [56]. The results of descriptive sensory analysis of raw beef samples are shown in Figure 1.

Initially, all samples were rated highly by the evaluators in all criteria (odor, color, overall acceptability). Only the samples CH-SMEO 0.5% and 1% were given a slightly lower rating for the odor, due to the fact that the EO has an intense odor. The limit grade of acceptability of the evaluated beef samples was 3 [24]. The control on the 4th day of storage was rated as borderline acceptable, and the same is the case of the CH samples, with a slight difference in the rating (*p* < 0.05). Compared to the control and CH samples, the addition of free EO provided extended freshness to the beef patties, up to the 8th day of storage. The use of nanoencapsulated EO had a better effect on the evaluated sensory characteristics of the beef. First, with the nanoencapsulation of the EO, the intense odor of the EO was significantly reduced. In this regard, it was noted that the samples treated only with 1% CH-SMEO-CNPs retained a satisfactory intensity of color and smell, even on the 16th day of storage, i.e., they were above the acceptable limits. The overall acceptability of beef samples supplemented with nanoencapsulated EO was extended by 4 days, compared to free EO, and by 12 days compared to the control.

Similarly, the use of *Paulownia Tomentosa* EO CNPs contributed to obtaining acceptable scores for the odor, color and overall acceptability of pork meat until the end of the observed storage period (16 days), compared to free EO [24]. Similar results were observed when using free and nanocapsulated tarragon EO for same storage period [30]. In lamb meat, *Satureja khuzestanica* EO and CNPs had a favorable effect on color stability and the appearance of unpleasant odors during the storage period of 20 days [22]. The use of free cinnamon EO extended the sensory acceptability of turkey meat up to 12 days of storage [34].

The effect of EO CNPs on prolonging the freshness of raw beef samples is explained, first of all, by the elimination of strong odors originating from EO. In this way, the EO is prevented from evaporating and giving the meat a strange aroma. Then, by means of nanoencapsulation, the EO particles remain incorporated inside the inert polymer, which enables their gradual and controlled release during the storage period and a constant effect on improving the sensory characteristics of the meat [11].

## 4. Conclusions

According to all performed analysis, all applied coatings had a positive impact on the microbiological and oxidative stability of the beef, compared to the control samples. By using pure chitosan coating, as well as coating with SMEO in concentrations of 0.25% and 0.5%, the shelf life of beef was extended by at least 2 days, compared to the control. Furthermore, the effect of the coating enriched with SMEO in a concentration of 1% extends the shelf life of beef for a minimum of 8 days, as well as the application of coatings with SMEO-CNPs in a concentration of 0.25% and 0.5%. In this case, SMEO-CNPs can be more acceptable because of the better scores in the sensory evaluation due to the neutralization of the strong smell of SMEO. The application of 1% SMEO-CNPs had the most pronounced effect on extending the shelf life of beef and keeping it fresh for at least 12 days, which is 5 times longer than in the case of the control sample. Successful nanoencapsulation of SMEO into chitosan nanoparticles resulted in the prolongation of microbiological stability as well as the slower oxidation of meat samples, delaying the process of color change and unpleasant odor formation. Good control of microbial growth and a reduced rate of oxidative changes, as well as good evaluation ratings, may indicate the potential application of chitosan coatings enriched with SMEO-CNPs in the meat industry.

## Figures and Tables

**Table 1:** Description of different treatments in present research.

	Treatment	Description
1	C	Control beef sample, with no treatment
2	CH	Beef treated with a chitosan coating
3	CH-SMEO 0.25%	Beef treated with a chitosan coating enriched with 0.25% SMEO
4	CH-SMEO 0.5%	Beef treated with a chitosan coating enriched with 0.5% SMEO
5	CH-SMEO 1%	Beef treated with a chitosan coating enriched with 1% SMEO
6	CH-SMEO-CNPs 0.25%	Beef treated with a chitosan coating enriched with 0.25% SMEO-CNPs
7	CH-SMEO-CNPs 0.5%	Beef treated with a chitosan coating enriched with 0.5% SMEO-CNPs
8	CH-SMEO-CNPs 1%	Beef treated with a chitosan coating enriched with 1% SMEO-CNPs

**Table 2:** Nanoparticle characterization parameters.

Sample *	Z-Average (d.nm)	Polydispersity Index (PDI)	Encapsulation Efficiency (%)
CNPs	415.4 ± 8.0 ^a^	0.718 ^a^	-
SMEO-CNPs	139.9 ± 2.8 ^b^	0.241 ^b^	63.2 ± 2.2

* CNPs—chitosan nanoparticles, SMEO-CNPs—chitosan-based nanoparticles loaded with *Satureja montana* L. essential oil. Different letters indicate significant difference among the samples in the same column.

**Table 3:** Effect of the addition of chitosan coatings and chitosan-based nanoparticles enriched with *Satureja montana* L. essential oil on the change in the total viable counts (TVC) in beef samples during storage.

Storage Period (Day)	C	Chitosan Coatings *						
		CH	CH-SMEO 0.25%	CH-SMEO 0.5%	CH-SMEO 1%	CH-SMEO-CNPs 0.25%	CH-SMEO-CNPs 0.5%	CH-SMEO-CNPs 1%
	log CFU/g							
**0**	2.35 ± 0.11 ^a, A^	2.41 ± 0.11 ^a, A^	2.38 ± 0.06 ^a, A^	2.46 ± 0.12 ^a, A^	2.43 ± 0.09 ^a, A^	2.36 ± 0.04 ^a, AB^	2.34 ± 0.04 ^a, A^	2.38 ± 0.03 ^a, A^
**2**	2.46 ± 0.01 ^a, A^	2.41 ± 0.01 ^b, A^	2.38 ± 0.01 ^b, A^	2.34 ± 0.01^c, A^	2.32 ± 0.07 ^c, A^	2.31 ± 0.01 ^cd, AB^	2.29 ± 0.07 ^de, A^	2.27 ± 0.03 ^e, A^
**4**	3.59 ± 0.06 ^a, B^	3.24 ± 0.02 ^b, B^	2.93 ± 0.07 ^c, B^	2.75 ± 0.03 ^d, B^	2.67 ± 0.06 ^d, B^	2.42 ± 0.01 ^e, B^	2.31 ± 0.04 ^ef, A^	2.28 ± 0.01 ^f, A^
**8**	5.95 ± 0.03 ^a, C^	5.55 ± 0.03 ^b, C^	4.22 ± 0.01 ^c, C^	4.12 ± 0.01 ^c, C^	3.93 ± 0.07 ^d, C^	3.30 ± 0.06 ^e, C^	2.96 ± 0.03 ^f, B^	2.39 ± 0.05 ^f, A^
**12**	7.09 ± 0.07 ^a, D^	6.61 ± 0.01 ^b, D^	6.48 ± 0.16 ^b, D^	5.88 ± 0.03 ^c, D^	5.22 ± 0.01 ^d, D^	4.96 ± 0.01 ^e, D^	4.45 ± 0.11 ^f, C^	3.00 ± 0.02 ^f, B^
**16**	7.61 ± 0.34 ^a, E^	6.83 ± 0.34 ^b, D^	6.72 ± 0.34 ^b, E^	6.62 ± 0.34 ^b, E^	6.13 ± 0.34 ^c, E^	5.88 ± 0.34 ^c, E^	5.76 ± 0.34 ^c, D^	5.28 ± 0.34 ^d, C^
**20**	8.04 ± 0.05 ^a, F^	7.36 ± 0.07 ^b, E^	6.79 ± 0.08 ^c, E^	6.74 ± 0.03 ^c, E^	6.54 ± 0.02 ^d, F^	6.16 ± 0.03 ^e, F^	5.99 ± 0.03 ^f, E^	5.64 ± 0.08 ^g, D^

* CH—chitosan coating; CH-SMEO—chitosan coating enriched with of *Satureja montana* L. essential oil in different concentrations; CH-SMEO-CNPs—chitosan coating enriched with different concentrations of chitosan-based nanoparticles loaded with *Satureja montana* L. essential oil; C—control sample. Different letters indicate significant difference among the samples in the same column (A–F) and row (a–g).

**Table 4:** Effect of the addition of chitosan coatings and chitosan-based nanoparticles enriched with *Satureja montana* L. essential oil on the change in the number *of Pseudomonas* spp. in beef samples during storage.

Storage Period (Day)	C		Chitosan Coatings *						
			CH	CH-SMEO 0.25%	CH-SMEO 0.5%	CH-SMEO 1%	CH-SMEO-CNPs 0.25%	CH-SMEO-CNPs 0.5%	CH-SMEO-CNPs 1%
	log CFU/g								
**0**	2.34 ± 0.11 ^a, A^	2.37 ± 0.09 ^a, A^		2.36 ± 0.11 ^a, A^	2.34 ± 0.06 ^a, A^	2.34 ± 0.12 ^a, A^	2.31 ± 0.11 ^a, A^	2.31 ± 0.06 ^a, A^	2.38 ± 0.12 ^a, A^
**2**	2.53 ± 0.02 ^a, A^	2.46 ± 0.09 ^ab, A^		2.41 ± 0.09 ^abc, AB^	2.37 ± 0.09 ^bcd, AB^	2.35 ± 0.09 ^bcd, A^	2.30 ± 0.09 ^cd, A^	2.24 ± 0.09 ^d, A^	1.75 ± 0.09 ^e, B^
**4**	2.85 ± 0.05 ^a, B^	2.76 ± 0.01 ^b, B^		2.53 ± 0.02 ^c, B^	2.41 ± 0.05 ^d, A^	2.37 ± 0.07 ^de, A^	2.32 ± 0.02 ^e, A^	2.30 ± 0.06 ^e, A^	2.18 ± 0.03 ^f, A^
**8**	5.45 ± 0.05 ^a, C^	4.60 ± 0.05 ^b, C^		3.95 ± 0.02 ^c, C^	3.45 ± 0.03 ^d, B^	3.00 ± 0.04 ^e, B^	2.97 ± 0.01 ^e, B^	2.78 ± 0.01 ^f, B^	2.69 ± 0.17 ^f, C^
**12**	6.65 ± 0.02 ^a, D^	6.13 ± 0.01 ^b, D^		5.53 ± 0.01 ^c, D^	5.13 ± 0.01 ^d, C^	4.95 ± 0.05 ^de, C^	4.84 ± 0.10 ^e, C^	4.47 ± 0.13 ^f, C^	4.25 ± 0.04 ^g, D^
**16**	7.01 ± 0.09 ^a, E^	6.83 ± 0.02 ^b, E^		5.86 ± 0.02 ^c, E^	5.67 ± 0.09 ^d, D^	5.25 ± 0.02 ^e, D^	5.18 ± 0.05 ^e, D^	5.03 ± 0.08 ^f, D^	4.38 ± 0.01 ^g, D^
**20**	7.79 ± 0.18 ^a, F^	6.94 ± 0.04 ^b, E^		6.16 ± 0.02 ^c, F^	5.97 ± 0.01 ^cd, E^	5.72 ± 0.01 ^de, E^	5.66 ± 0.03 ^e, E^	5.24 ± 0.10 ^f, D^	4.77 ± 0.20 ^g, E^

* CH—chitosan coating; CH-SMEO—chitosan coating enriched with of *Satureja montana* L. essential oil in different concentrations; CH-SMEO-CNPs—chitosan coating enriched with different concen-trations of chitosan-based nanoparticles loaded with *Satureja montana* L. essential oil; C—control sample. Different letters indicate significant difference among the samples in the same column (A–F) and row (a–g).

**Table 5:** Effect of the addition of chitosan coatings and chitosan-based nanoparticles enriched with *Satureja montana* L. essential oil on the change in the number of lactic acid bacteria (LAB) in beef samples during storage.

Storage Period (Day)	C	Chitosan Coatings *						
		CH	CH-SMEO 0.25%	CH-SMEO 0.5%	CH-SMEO 1%	CH-SMEO-CNPs 0.25%	CH-SMEO-CNPs 0.5%	CH-SMEO-CNPs 1%
	log CFU/g							
**0**	1.47 ± 0.01 ^a, A^	1.41 ± 0.23 ^a, A^	1.34 ± 0.11 ^a, A^	1.44 ± 0.44 ^a, A^	1.33 ± 0.09 ^a, A^	1.41 ± 0.23 ^a, A^	1.41 ± 0.23 ^a, AB^	1.41 ± 0.23 ^a, AC^
**2**	1.77 ± 0.01 ^a, A^	1.60 ± 0.05 ^b, A^	1.78 ± 0.06 ^a, B^	1.75 ± 0.04 ^a, A^	1.70 ± 0.01 ^a, B^	1.15 ± 0.01 ^c, B^	1.12 ± 0.05 ^c, A^	1.11 ± 0.04 ^c, B^
**4**	2.75 ± 0.01 ^a, B^	2.34 ± 0.15 ^b, B^	2.37 ± 0.03 ^b, C^	2.36 ± 0.06 ^b, B^	2.06 ± 0.03 ^c, C^	1.74 ± 0.01 ^d, C^	1.59 ± 0.05 ^d, BC^	1.28 ± 0.06 ^e, A^
**8**	3.44 ± 0.18 ^a, C^	2.84 ± 0.05 ^b, C^	2.80 ± 0.08 ^b, D^	2.59 ± 0.06 ^bcB^	2.32 ± 0.18 ^c, D^	2.01 ± 0.06 ^d, D^	1.83 ± 0.04 ^de, C^	1.66 ± 0.03 ^e, C^
**12**	5.26 ± 0.022 ^a, D^	4.92 ± 0.03 ^b, D^	4.64 ± 0.05 ^b, E^	4.30 ± 0.04 ^c, C^	4.22 ± 0.09 ^c, E^	2.06 ± 0.05 ^d, D^	3.00 ± 0.29 ^e, D^	2.11 ± 0.08 ^d, D^
**16**	6.38 ± 0.23 ^a, E^	5.57 ± 0.05 ^b, E^	5.24 ± 0.02 ^c, F^	4.82 ± 0.06 ^d, D^	4.77 ± 0.07 ^d, F^	4.35 ± 0.03 ^e, E^	4.01 ± 0.01 ^f, E^	3.09 ± 0.02 ^g, E^
**20**	6.35 ± 0.01 ^a, E^	5.88 ± 0.02 ^b, E^	5.80 ± 0.06 ^b, G^	4.88 ± 0.13 ^c, D^	4.75 ± 0.09 ^c, F^	4.24 ± 0.02 ^d, E^	4.07 ± 0.03 ^d, E^	3.81 ± 0.06 ^e, F^

* CH—chitosan coating; CH-SMEO—chitosan coating enriched with of *Satureja montana* L. essential oil in different concentrations; CH-SMEO-NPs—chitosan coating enriched with different concentrations of chitosan-based nanoparticles loaded with *Satureja montana* L. essential oil; C—control sample. Different letters indicate significant difference among the samples in the same column (A–G) and row (a–g).

**Table 6:** Effect of the addition of chitosan coatings and chitosan-based nanoparticles enriched with *Satureja montana* L. essential oil on the change in the number of psychrophilic bacteria in beef samples during storage.

Storage Period (Day)	C	Chitosan Coatings *						
		CH	CH-SMEO 0.25%	CH-SMEO 0.5%	CH-SMEO 1%	CH-SMEO-CNPs 0.25%	CH-SMEO-CNPs 0.5%	CH-SMEO-CNPs 1%
	log CFU/g							
**0**	2.34 ± 0.08 ^a, A^	2.47 ± 0.08 ^a, A^	2.43 ± 0.33 ^a, A^	2.44 ± 0.08 ^a, A^	2.45 ± 0.11 ^a, A^	2.43 ± 0.33 ^a, A^	2.44 ± 0.08 ^a, A^	2.45 ± 0.11 ^a, A^
**4**	3.34 ± 0.02 ^a, B^	3.21 ± 0.03 ^b, B^	3.11 ± 0.08 ^c, B^	2.91 ± 0.01 ^d, B^	2.75 ± 0.01 ^e, B^	2.66 ± 0.02 ^f, A^	2.60 ± 0.07 ^g, A^	2.54 ± 0.01 ^h, A^
**8**	5.66 ± 0.03 ^a, C^	5.22 ± 0.01 ^b, C^	4.37 ± 0.05 ^c, C^	4.22 ± 0.02 ^d, C^	4.07 ± 0.04 ^e, C^	3.33 ± 0.04 ^f, B^	3.16 ± 0.08 ^g, B^	2.94 ± 0.05 ^h, B^
**12**	6.97 ± 0.02 ^a, D^	6.75 ± 0.04 ^aD^	6.64 ± 0.08 ^b, D^	5.94 ± 0.04 ^c, D^	5.31 ± 0.18 ^d, D^	4.98 ± 0.01 ^e, C^	4.86 ± 0.07 ^e, C^	4.43 ± 0.18 ^f, C^
**16**	7.13 ± 0.03 ^a, E^	6.89 ± 0.01 ^b, D^	6.76 ± 0.01 ^c, D^	6.43 ± 0.01 ^d, E^	6.12 ± 0.04 ^e, E^	5.92 ± 0.07 ^f, D^	5.85 ± 0.02 ^f, D^	5.10 ± 0.07 ^g, D^
**20**	7.84 ± 0.03 ^a, F^	7.28 ± 0.04 ^b, E^	6.75 ± 0.13 ^c, D^	6.70 ± 0.01 ^c, F^	6.47 ± 0.01 ^d, F^	6.01 ± 0.04 ^e, D^	5.80 ± 0.08 ^f, D^	5.35 ± 0.03 ^g, D^

* CH—chitosan coating; CH-SMEO—chitosan coating enriched with of *Satureja montana* L. essential oil in different concentrations; CH-SMEO-CNPs—chitosan coating enriched with different concentrations of chitosan-based nanoparticles loaded with *Satureja montana* L. essential oil; C—control sample. Different letters indicate significant difference among the samples in the same column (A–F) and row (a–g).

**Table 7:** Change in pH value in beef samples treated with chitosan coatings and chitosan-based nanoparticles with the addition of *Satureja montana* L. essential oil.

Storage Period (Day)	C	Chitosan Coatings *						
		CH	CH-SMEO 0.25%	CH-SMEO 0.5%	CH-SMEO 1%	CH-SMEO-CNPs 0.25%	CH-SMEO-CNPs 0.5%	CH-SMEO-CNPs 1%
**0**	5.49 ± 0.07 ^a, A^	5.24 ± 0.09 ^b, A^	5.45 ± 0.05 ^ac, A^	5.33 ± 0.11 ^abc, A^	5.29 ± 0.04 ^bc, A^	5.38 ± 0.03 ^abc, A^	5.39 ± 0.06 ^abc, A^	5.39 ± 0.02 ^abc, A^
**2**	5.59 ± 0.08 ^ac, A^	5.56 ± 0.06 ^abc, B^	5.65 ± 0.03 ^a, B^	5.49 ± 0.07 ^cd, AB^	5.43 ± 0.02 ^bd. B^	5.45 ± 0.02 ^bd, A^	5.42 ± 0.03 ^d, A^	5.40 ± 0.01 ^d, A^
**4**	5.83 ± 0.06 ^a, B^	5.73 ± 0.05 ^ab, CD^	5.71 ± 0.06 ^b, C^	5.57 ± 0.05 ^abc, B^	5.48 ± 0.03 ^e, B^	5.63 ± 0.05 ^bcd, A^	5.61 ± 0.04 ^bcde, B^	5.50 ± 0.01 ^de, B^
**8**	6.10 ± 0.02 ^a, C^	5.98 ± 0.02 ^ab, CD^	5.90 ± 0.01 ^b, C^	5.88 ± 0.11 ^b, C^	5.67 ± 0.05 ^c, C^	5.72 ± 0.01 ^c, B^	5.68 ± 0.05 ^c, B^	5.51 ± 0.01 ^d, B^
**12**	6.83 ± 0.02 ^a, D^	6.26 ± 0.05 ^b, D^	6.18 ± 0.03 ^bc, D^	6.15 ± 0.03 ^cd, D^	6.10 ± 0.01 ^cd, D^	6.08 ± 0.01 ^d, C^	5.92 ± 0.03 ^e, C^	5.72 ± 0.03 ^f, C^
**16**	6.93 ± 0.06 ^a, D^	6.77 ± 0.22 ^ab, E^	6.75 ± 0.12 ^ab, E^	6.46 ± 0.03 ^cd, E^	6.29 ± 0.08 ^cd, E^	6.53 ± 0.05 ^bc, D^	6.33 ± 0.04 ^cd, D^	6.18 ± 0.03 ^d, D^
**20**	7.13 ± 0.01 ^a, E^	6.84 ± 0.07 ^b, E^	6.77 ± 0.09 ^b, E^	6.72 ± 0.01 ^bc, F^	6.71 ± 0.02 ^bc, F^	6.60 ± 0.09 ^cd, D^	6.54 ± 0.03 ^d, E^	6.29 ± 0.06 ^e, E^

* CH—chitosan coating; CH-SMEO—chitosan coating enriched with of *Satureja montana* L. essential oil in different concentrations; CH-SMEO-CNPs—chitosan coating enriched with different concentrations of chitosan-based nanoparticles loaded with *Satureja montana* L. essential oil; C—control sample. Different letters indicate significant difference among the samples in the same column (A–F) and row (a–f).

**Table 8:** Malonaldehyde (MDA) content in beef samples treated with chitosan coatings and chitosan-based nanoparticles enriched with *Satureja montana* L. essential oil during storage.

Storage Period (Day)	C	Chitosan Coatings *						
		CH	CH-SMEO 0.25%	CH-SMEO 0.5%	CH-SMEO 1%	CH-SMEO-CNPs 0.25%	CH-SMEO-CNPs 0.5%	CH-SMEO-CNPs 1%
	mg MDA/kg							
**0**	0.31 ± 0.03 ^a, A^	0.28 ± 0.08 ^a, A^	0.31 ± 0.05 ^a, A^	0.29 ± 0.09 ^a, A^	0.31 ± 0.08 ^a, A^	0.27 ± 0.06 ^a, A^	0.32 ± 0.05 ^a, A^	0.29 ± 0.08 ^a, AB^
**2**	0.76 ± 0.17 ^a, AB^	0.66 ± 0.07 ^ab, B^	0.47 ± 0.01 ^bc, A^	0.40 ± 0.04 ^cd, A^	0.30 ± 0.05 ^cde, A^	0.33 ± 0.03 ^cde, AB^	0.21 ± 0.01 ^de, B^	0.19 ± 0.01 ^e, A^
**4**	0.91 ± 0.08 ^a, B^	0.80 ± 0.10 ^a, BC^	0.48 ± 0.02 ^b, A^	0.46 ± 0.03 ^b, A^	0.41 ± 0.07 ^b, A^	0.40 ± 0.01 ^b, AB^	0.37 ± 0.01 ^b, A^	0.34 ± 0.02 ^b, AB^
**8**	1.35 ± 0.06 ^a, BC^	0.94 ± 0.02 ^b, BC^	0.55 ± 0.09 ^c, AB^	0.49 ± 0.06 ^cd, A^	0.47 ± 0.08 ^cd, AB^	0.44 ± 0.06 ^cde, B^	0.36 ± 0.02 ^de, A^	0.28 ± 0.05 ^e, AB^
**12**	1.60 ± 0.06 ^a, C^	1.04 ± 0.06 ^b, C^	0.94 ± 0.09 ^b, BC^	0.76 ± 0.03 ^c, B^	0.63 ± 0.03 ^cd, B^	0.60 ± 0.06 ^cd, C^	0.57 ± 0.03 ^d, C^	0.47 ± 0.03 ^d, BC^
**16**	1.69 ± 0.08 ^a, C^	0.97 ± 0.10 ^b, BC^	1.05 ± 0.08 ^b, C^	0.93 ± 0.18 ^bc, B^	1.00 ± 0.03 ^b, C^	0.80 ± 0.09 ^cd, D^	0.68 ± 0.02 ^cd, D^	0.54 ± 0.03 ^d, CD^
**20**	3.26 ± 0.51 ^a, D^	3.09 ± 0.25 ^a, D^	2.62 ± 0.35 ^a, D^	1.38 ± 0.05 ^b, C^	1.10 ± 0.10 ^b, C^	0.90 ± 0.03 ^b, D^	0.81 ± 0.04 ^b, E^	0.69 ± 0.14 ^b, D^

* CH—chitosan coating; CH-SMEO—chitosan coating enriched with of *Satureja montana* L. essential oil in different concentrations; CH-SMEO-CNPs—chitosan coating enriched with different concentrations of chitosan-based nanoparticles loaded with *Satureja montana* L. essential oil; C—control sample. Different letters indicate significant difference among the samples in the same column (A–E) and row (a–e).

## Data Availability

Data is contained within the article.
